# Vitamin A supplementation coverage and its associated factors among children aged 6–59 months in West Azernet Berbere Woreda, South West Ethiopia

**DOI:** 10.1186/s12887-023-04059-1

**Published:** 2023-05-23

**Authors:** Bihon Berihun, Fantaye Chemir, Mehari Gebru, Fisha Alebel GebreEyesus

**Affiliations:** 1 Lera Health Center, Siltie, Ethiopia; 2 Department of Midwifery, College of Medicine and Health Sciences, Wolkite University, PO Box 07, Wolkite, Ethiopia; 3 Department of Public Health, College of Medicine and Health Sciences, Wolkite University, PO Box 07, Wolkite, Ethiopia; 4 Department of Nursing, College of Medicine and Health Sciences, Wolkite University, PO Box 07, Wolkite, Ethiopia

**Keywords:** Vitamin A, Coverage, Supplementation, West Azernet Berbere Woreda, Ethiopia

## Abstract

**Background:**

Vitamin A deficiency is one of the major public health problems in low and middle-income countries including Ethiopia. Despite this fact, little attention was given to routine vitamin A supplementation in hard-to-reach rural areas and districts. Therefore, this study aimed to assess vitamin A supplementation coverage and its associated factors among children aged 6–59 months in West Azernet Berbere woreda, southern Ethiopia, 2021.

**Methods:**

A community-based cross-sectional study was conducted from April to May 2021. A total sample size of 471 study participants was involved in the study area. A simple random sampling technique was used to recruit the study subject. A pretested structured interviewer-administered questionnaire was used. Bivariable and multivariable logistic regression analyses were done to identify variables having a significant association with vitamin A supplementation. The variables having a *p*-value ≤ 0.05 with 95% CI were used to declare an association between factors and a dependent variable.

**Results:**

In this study, a total of 471 respondents were successfully interviewed with a response rate of 97.3%. The coverage of vitamin A supplementation was found to be 58.0%. Family monthly income [AOR = 2.565, 95% CI(1.631,4.032)], having PNC visit [AOR = 1.801, 95% CI (1.158, 2.801)], husbands disapproval about vitamin A supplementation [AOR = 0.324, 95% CI (0.129, 0.813)], information about vitamin A supplementation [AOR = 2.932, 95% CI (1.893, 4,542)] and ANC follow-up [AOR = 1.882, 95% CI (1.084, 3.266)] were factors significantly associated to vitamin A supplementation.

**Conclusion:**

Vitamin A supplementation was found to be low and it is strongly associated with family monthly income, postnatal care, husband's disapproval of vitamin A supplementation, antenatal care follow-up, and information about vitamin A supplementation. Based on our findings, it is recommended to improve the monthly income of the household by actively engaging in various income-generating activities, enhance health information dissemination among mothers, particularly those who are underprivileged by using different strategies like local health campaigns, and mass media, advocacy of antenatal, and postnatal follow-up and promote the involvement of males/husband in childhood immunization service.

## Background

Vitamin A is a fat-soluble compound that can be categorized into two classes which are found in animal food sources (preformed vitamin A or retinol) and fruit and vegetable sources (pro-vitamin carotenoid) [1]. It plays a fundamental role in numerous physiological functions including vision, immunity, red blood cell production, and growth [2, 3]. Since 2000, the dramatic scale-up of VAS programs has protected millions of children from the devastating consequences of vitamin A deficiency. Yet today, VAS programs are in crisis [4, 5]. For instance, recent data showed that more than 140 million children were left behind, putting them at risk of disease and death [6]. Vitamin A deficiency (VAD)- serum retinol level < 0.70 μmol/l is one of the most prevalent micronutrient deficiencies in the world [7]. According to a report by UNICEF (2015), VAD is the third most widespread and common serious nutritional disorder among young children after protein-energy malnutrition and anemia caused by iron deficiency [8].

Children under five years and Women of Reproductive Age (WRA) are the greatest vulnerable groups at risk for VAD [7]. To this end, the WHO recommends semi-annual delivery of two doses of 200,000 IU of vitamin A to children 1–5 years of age and one dose of 100,000 IU for infants 6–11 months in countries where vitamin A deficiency is recognized as a public health problem [9–11].

Globally, about 3 in 10 children under the age of 5 years are vitamin A deficient, and an estimated 2% of all deaths are attributable to VAD in this age group [4, 12]. Moreover, around 650,00 early childhood deaths from diarrhea, measles, malaria, and other infections each year are contributed to VAD as an underlying cause [13]. In Africa, an estimated 44.4% of preschool children have Vitamin A deficiency and 2% of preschool-age children are affected by night blindness, which is four times higher than the proportion in South East Asia (0.5%) [14, 15]. In Ethiopia, the prevalence of vitamin A deficiency is one of the significant public health problems. It leads to 80,000 deaths in a year and affected 61% of under-five children [16, 17].

Ethiopia has been implementing periodic administration of high-dose vitamin A oral supplements through the campaign-based, vertical Enhanced Outreach Strategy (EOS) since 2004 [18]. According to World Bank data, in Ethiopia, vitamin A supplementation remained above 80% from 2006 to 2011. However, after 2012 which coincides with a shift from a campaign-based approach the coverage dropped below 80% [2]. To avert this figure the Ethiopian Ministry of Health adopted various policies and strategies to enhance vitamin A coverage using enhanced outreach strategy, community health days, the routine health extension program, and periodic supplementation to reduce morbidity, mortality, and blindness among the children in Ethiopia [2, 19]. Despite these measures being considered to enhance vitamin A supplementation coverage, vitamin A deficiency remained a major problem for decades [20]. A recent report from the global nutrition report showed that the coverage of two high-dose vitamin A supplements was 66% by 2020 [21] which is by far less than the Health Sector Transformation Plan I (HSTP I) goal of increasing the proportion of children aged 6–59 months who receive vitamin A supplementation to 95% by the end of 2020 [22].

VAD among children is associated with increased child mortality mainly due to detrimental effects on the immune system and it is associated with various poor social, economic, and ecological conditions [23, 24]. VAD is high in rural areas and varied significantly with the season, ethnicity, region, and vaccination status [25]. Moreover, the uptake of vitamin A supplementation is influenced by socio-economic, demographic, and geographical factors. Such as ANC follow-up [26, 27] parental educational status [12, 28, 29], maternal knowledge [2, 30], monthly income and wealth index [2, 12, 29], maternal employment status [27, 29], media exposure [27–29], age of the child [12, 28–31], place of delivery [27], and distance to the health institution [30, 32].

Studies conducted in Ethiopia have established the fact that Vitamin A deficiency is a major public health problem [2, 30, 33]. The vitamin A supplementation program is still the main choice in dealing with vitamin A deficiency cases. However, there is still a need for other efforts in the future to achieve sustainable prevention: among these improving infant and young child feeding practices, and nutrition education. Vitamin A fortification in food sources and nutrition-focused agricultural programs were mentioned [34–36]. A more recent study carried out in Ethiopia showed that there are extensive socioeconomic and geographic-based disparities in VAS coverage across regions and districts [37, 38]. So, this study aimed to explore vitamin A supplementation coverage and its associated factors among children aged 6–59 months in West Azernet Berber Woreda, southern Ethiopia, 2021. The findings of the study will provide paramount significance to children to prevent the negative consequences of vitamin A deficiency by identifying the factors that affect the utilization of VAS in the study area.

## Methods and materials

### Study area and period

West Azernet Berbere Woreda is found 267 km away from Addis Ababa, the capital city of Ethiopia in the southwest direction along the main Addis Ababa to Hosanna road, and 274 km from the capital city of the SNNP region of Hawassa town. According to the West Azernet Berbere Woreda town city administration health office report in 2020, the population size of the town is estimated to be 83, 101 and from these 40, 719 males, 42, 382 are females, infants 2,651and children 6–59 months of age 10,024. West Azernet Berbere Woreda had 4 health centers, 2 urban and 2 rural, and 18 rural and 1 urban health posts. The health facilities found in the district provide preventive, curative, and rehabilitative health care services for the population residing in West Azernet Berbere woreda and other nearby districts and also serve as referral centers and practical training sites for health extension workers. Similarly, health posts provide various preventive and health promotion services, in addition to treating cases such as malaria, pneumonia, scabies, trachoma, and other mild illnesses. Moreover, these health facilities provide immunization services and vitamin A supplementation based on the national EPI guidelines. The study was conducted from April to May 2021.

### Study design

A community-based cross-sectional study design was conducted.

#### Population

The source population was all mothers of children aged 6–59 months in West Azernet Berbere Woreda and the study population was randomly selected mothers of children aged 6–59 months who reside in West Azernet Berbere Woreda.

### Inclusion & exclusion criteria

All mothers of children aged between 6–59 months who reside greater than 6 months were included in the study whereas mothers who were seriously ill and had difficulty communicating were excluded from the study.

#### Sample size

We have determined the sample size using the single population proportion formula and using variables that have a statistically significant association with the outcome variables in previously published articles then we use the variables which yield the maximum sample size.

1. Sample size determination for the first specific objective

The sample size for this study was determined by using a single population proportion formula in Ethiopia. [*n* = [(Za/2)^2^.P (1-P)]/d^2^] by assuming a 95% confidence level (Z a/2 = 1.96), a margin of error of 5%, P = proportion of vitamin-supplementation coverage in Humbo district, Southern Ethiopia (75%) [39]. And a 10% addition for the non-response rate. The final sample size will be 317.

2. Sample size calculation for the second specific objective

The sample size is calculated by using simple random sampling formula using Epi info version 7 with the assumption of a 95% confidence interval, a ratio of exposed to unexposed one, and a power of 80%. We have used knowledge of VAS (AOR = 1.49), the educational status of the respondents (AOR = 0.53), and the wealth index (AOR = 1.80) [39]. From these variables, educational status yields the maximum sample size which is 484. The total sample size was compared between for first and second specific objectives and the largest sample size of 484 was taken including a 10% none response rate.

### Sampling technique and procedure

A simple random sampling technique was used to employ the required study participants. In this study, all four clusters namely the Lera cluster, Mugo cluster, Jiro cluster, and Bilalo cluster were included in the study. Each cluster incorporates a minimum of four and a maximum of seven "kebeles" (i.e., the lowest governmental administrative unit in Ethiopia). The first total sample size was proportionally allocated to each cluster (Lera cluster (149), Mugo cluster (120), Jiro cluster (78), and Bilalo cluster (124), based on the total number of women having an eligible child in each cluster, then we used the registered data from the health extension workers before data collection to provide an identity code for each eligible household in all clusters. After addressing the number of mothers having eligible children a simple random sampling technique was used in each cluster separately.

### Variables

#### Dependent variable

- Vitamin A supplementation (yes/no)

##### Independent variables

**Socio-demographic and socioeconomic characteristics**

Status of the caregiver, maternal age, residence, religion, ethnicity, maternal educational level, current maternal occupation, marital status.

**Maternal healthcare-related characteristics**

Current father's occupation, decision maker of the household, family income, family size, number of under-five children, child age, time taken to get to the nearest health institution, ANC follow-up, place of delivery, PNC follow-up, nutritional counseling, advice from peers and fathers approval.

**Maternal knowledge regarding VA and Accessibility of VAS**

Maternal knowledge regarding vitamin-AS, the importance of VAS**,** Schedule, Media Exposure.

**Child-related characteristics**

History of growth monitoring follow-up**,** child nutritional status (MUAC)**.** Child dietary diversity score, birth order of the child**,** childhood illness (Diarrhea, AURTI, pneumonia, malnutrition).

### Operational definition

**Vitamin-A-supplementation (Yes/No)** Yes = if the child took 100,000 IU below 12 months and 200,000 IU greater than 12 months of vitamin-A supplementation, No = if the child did not take VAS at all by showing the vitamin A capsule.

**Information provision about Vitamin A supplementation** is a process within which information is provided to mothers/clients without any individualization of content on the source of vitamin A, its function, age-specific dose, route, side effects of Vitamin A capsule, and about vitamin A deficiency and its public health importance.

**Knowledge about VAS (Poor/Good)**—There are four knowledge questions which score a total of eight; Poor = score of < 50%, Good = score of ≥ 50%

**VA supplemented child**; A child who was given a Vitamin A capsule in the preceding 6 months of the survey as reported by the mother (after the mother is shown the capsule) [33].

Mid-upper arm circumference (MUAC) is a measure to assess nutritional status. It is measured on a straight left arm, midway between the tip of the shoulder and the tip of the elbow. (Severe acute malnutrition, MUAC < 115 mm, moderate acute malnutrition, MUAC ≥ 115 mm and < 125 mm. at risk of malnutrition, MUAC ≥ 125 mm and < 135 mm, well nourished, MUAC ≥ 135 mm) [40, 41].

In this study, a minimum dietary diversity was defined as the percentage of children 6–23 months of age who consumed foods and beverages from at least five out of eight defined food groups during the previous day [42].

### Data collection procedure

A structured questionnaire was developed through a critical review of relevant literature. The questionnaire had four parts. These are socio-demographic and socioeconomic characteristics, maternal health-related characteristics, maternal knowledge regarding vitamin-A and VAS, and child healthcare-related characteristics.

Direct face-to-face interviewing was conducted with mothers or caregivers having children aged 6–59 months, by giving a code for that specific household, to make sure whether the child took VAS or not. EPI chart was used, for those who don’t have EPI chart maternal recall was considered. We have recruited eight diploma health professionals as data collectors and two BSc public health professionals as supervisors.

### Data quality control measures

The research questionnaire was prepared in the English version and translated into the local language (Amharic) and retranslated back to English to check consistency by experts. Before the actual data collection, the questionnaire was pre-tested on 10% (49 mothers) of those living in Kebul town which is out of the study area, and then necessary modification was done accordingly. To minimize recall bias error on whether the child took vitamin-A supplementation cross-checking with the other family member was done. Continuous supervision and follow-up of the data collectors were made to review and check for completeness and consistency of the collected data on daily bases by supervisors and principal investigators. Incomplete and unclearly filled questionnaires were given back to the interviewer and the interviewers were going back to the coded household and fulfilled the questionnaire by interviewing the mother. The collected data will be handled and stored carefully and appropriately.

### Data processing and analysis

Data were cleaned, coded, and entered into Epi-data version 3.1 then it was transferred into SPSS version 21 for data processing and analysis. Percentage, mean, and standard deviation were used to summarize the data. Bivariable and multivariable logistic regression analyses were done to identify variables having a significant association with vitamin A supplementation. Variables with a p-value less than 0.25 in the bivariable models were considered candidate variables for the multivariable analysis [39, 43]. For model fit, Hosmer and Lemeshow test was carried out and found to be (0.78) which indicated the final model was well fitted The multicollinearity effect among candidate variables was checked using variance inflation factor (VIF) and found to be < 5. Variables with a *P*-value of ≤ 0.05 and AOR 95% CI was considered as statistically significant.

### Ethical consideration

Ethical clearance was obtained from the Ethical Review Committee of Wolkite University, College of Medicine and health science, and it was offered to the West Azernet Berber Woreda town health office. The purpose and importance of the study were explained, and written informed consent was obtained from a parent and/or legal guardian for study participation. All the information taken from the respondents has been used for research purposes only. Confidentiality and privacy were maintained by omitting the name of the respondents.

## Results

### Socio-demographic and economic characteristics

In this study, a total of 471 caregivers participated with a cumulative response rate of 97.3%. The majority of the study participants (87.5%) were married. About three-fifths (59.4%) of maternal age was between 25–34 years with a mean age of 1.0318 ± 0.63668 SD and the Majority of the child (88.1%) age was between 12–59 months with a mean age of 1.8811 ± 0.32401 SD.

Around three-fourths of mothers (72.2%) were living in a rural area; around two-thirds of the mother (64.8%) was attending primary and secondary school. A round 6 into 10 (57.7%) mothers were housewives followed by 73(15.5%) of the mothers who were employed at governmental institutions. In addition to this, most of the time the household decision makers were either the husband alone (38.9%) or the mother and father jointly (40.3%), and considering, the number of under-five children, the majority of the participants (93.2%) had at least two eligible children. Regarding the economic status of the family 270(57.3%) had less than 2000 Ethiopian birr monthly income (Table 1).

**Table 1:** Socio-demographic characteristic of care givers who have children aged 6–59 months, in West Azernet Berbere woreda, south west Ethiopia, 2021

Variables	Frequency	Percentage (%)
**Child age**		
6–11	56	11.9
12–59	415	88.1
**Child sex**		
Male	256	54.4
Female	215	45.6
**Maternal age**		
15–24	88	18.7
25–34	280	59.4
35–49	103	21.9
**Maternal relation**		
Biological	467	99.2
Foster	4	0.8
**Residence**		
Rural	340	72.2
Urban	131	27.8
**Maternal education**		
No formal education	101	21.4
Primary and secondary	305	64.8
Tertiary education	60	12.7
**Maternal occupation**		
Student	23	4.9
Housewife	272	57.7
Government employee	73	15.5
Non-government employee	13	2.8
Merchant	85	18
Daily laborer	5	1.1
**Marital status**		
Married	412	87.5
Divorced	16	3.4
Widowed	11	2.3
Separated	32	6.8
**Fathers educational status**		
No formal education	38	9.2
Primary and secondary	242	58.7
Tertiary	127	30.8
Religious education	5	1.1
**Fathers occupation**		
Student	1	0.2
Government employee	121	29.4
Non-government employee	47	11.4
Merchant	93	22.6
Daily laborer	48	11.7
Farmer	102	24.8
**Monthly income**		
< 2000 ETB	270	57.3
≥ 2000 ETB	201	42.7
**Household decision maker**		
Husband	183	38.9
Wife	98	20.8
Jointly	190	40.3
**Family size**		
≤ 4	204	43.3
5–6	200	42.5
7–10	67	14.2
**Number of < 5 children**		
≤ 2	438	93.2
3–4	33	6.8
**Times take to health facility (minutes)**		
< 30	226	48
30–59	187	39.7
≥ 60	58	12.3

*ETB* Ethiopian Birr

### Maternal healthcare-related characteristics

In this study, the majority of the study participants (84.3%) were married between the ages of 15–20 years. And more than 90% of mothers delivered their last child at health institutions. Moreover, around four-fifths of the respondents (79.6%) had ≥ 3 ANC visits, 219(46.5%) had postnatal care, more than half (53.7%) got advice from peers/ family about vitamin A supplementation, and 331(70.3%) of mothers got nutrition related counseling by health workers. Whereas 34(7.2%) mothers were disapproved by their husbands about vitamin A supplementation, 267(57.4%) mothers deliver the index child above two years intervals (Table 2).

**Table 2:** Maternal health care related characteristics of care givers who have children aged 6–59 months, in West Azernet Berbere woreda, south west Ethiopia,2021

Variables	Frequency	Percentage (%)
**Number of pregnancy**		
≤ 3	312	66.2
4–6	147	31.2
7–9	12	2.5
**Alive child**		
≤ 3	337	71.5
4–6	126	26.8
7–9	8	1.7
**Age at marriage**		
15–20	397	84.3
21–25	67	14.2
26–30	7	1.5
**Age at first pregnancy**		
15–20	329	69.9
21–25	124	26.3
26–33	8	3.8
**ANC follow-up**		
Yes	436	92.6
No	35	7.4
**Number of ANC follow up**		
≤ 2	89	20.4
≥ 3	347	79.6
**Birth place**		
Health institution	428	90.9
Home	43	9.1
**Postnatal care visit**		
Yes	219	46.5
No	252	53.3
**Place of PNC**		
Home	41	18.7
Health institution	178	81.3
**Birth interval**		
< 23 month	201	42.6
≥ 24 month	270	57.4
**distance to health facility(km)**		
≤ 2.5	363	77.1
> 2.5	108	22.9
**Nutrition counseling by Health work**		
Yes	331	70.3
No	140	29.7
**Advice from peers/family about VAS**		
Yes	253	53.7
No	218	46.3
**Husbands disapproval**		
Yes	34	7.2
No	437	92.8
**Maternal work load**		
Yes	70	14.9
No	401	85.1

### Maternal knowledge-related characteristics

**Fig. 1:**
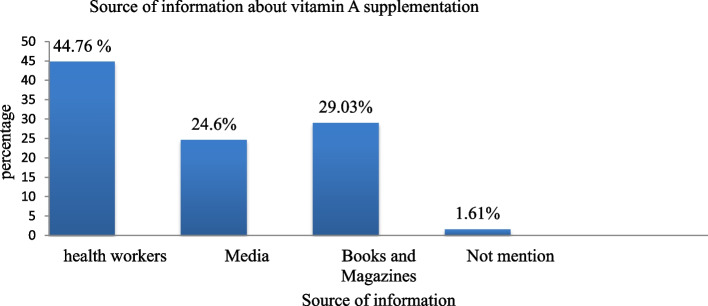
Source of information about vitamin A supplementation among mothers who had children aged 6–59 months in West Azernet Berbere Woreda South West Ethiopia, 2021 (*n* = 471)

Around three-fourths (74.7%) of the caregivers were ever heard about sources of vitamin A, and more than half of the participants (54.15) mentioned egg and milk as sources of VA, 37.4% mentioned vegetables and fruit whereas 8.5% of respondents didn't mention any food source and 170(48.2%) did not know the medical consequence of VAD whereas 37.1% of the respondent mentioned night blindness as a consequence of VAD (Table 3). Moreover, in this study 247(52.65%) mothers heard about vitamin A supplementation, from those mothers 44.76% of them heard from health workers, followed by 29.03% from books &magazines and 24.6% from media (Fig. 1). In this study 249(52.9%) mothers had good knowledge about vitamin A supplementation.

**Table 3:** Maternal knowledge related characteristics of care givers who have children aged 6–59 months, in West Azernet Berbere woreda, south west Ethiopia, 2021

Variables	Frequency	Percent (%)
**Have you heard VA source food**		
Yes	352	74.7
No	119	25.3
**Mention VA Food source**		
Not mention	30	8.5
Vegetable and fruits	132	37.4
Egg and milk	191	54.1
**Mention Medical Consequence of VAD**		
Not mention	170	48.2
Night blindness	131	37.1
Growth failure and skin dryness	52	14.7
**Over all knowledge on Vitamin A**		
Poor	222	47.1
Good	249	52.9

### Vitamin A supplementation coverage

**Fig. 2:**
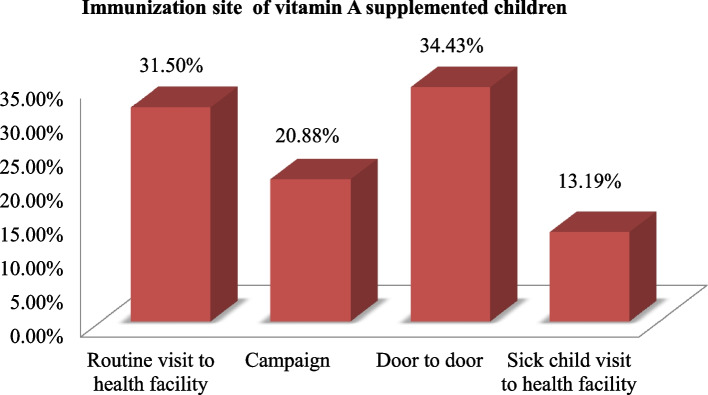
Immunization site of vitamin A-supplemented children in West Azernet Berbere Woreda, South West, Ethiopia, 2021 **(***n* = 471)

**Fig. 3:**
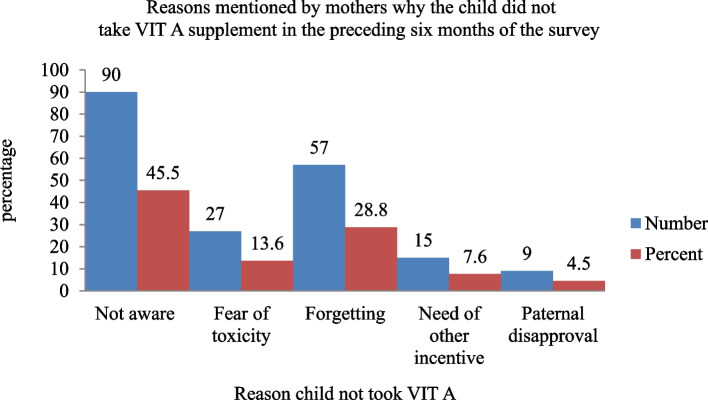
Reasons mentioned by mothers why the child did not take VIT A supplement in the preceding six months of the survey in West Azernet Berbere Woreda, 2021( *n* = 198)

The VAS status of the study children was determined in two ways, either from written vaccination records, i.e. the infant immunization card, or, from mothers' verbal reports. In this study, mothers were asked to show the interviewer the infant immunization card used to record the child's immunization status for VAS. If the infant immunization card was available, the interviewer copied the dates of the VAS received. If vaccination was not recorded in the infant immunization card, she was asked to recall whether the child had received VAS by showing the mother a vitamin A capsule and asking whether the child had received the same in the preceding 6 months of the survey relying on the mother's report. Accordingly, 58% of the mothers reported that their children received the capsule in the reference period. There was a significant variation among the age group in taking vitamin A capsules. Thirty-seven (13.6%) of the children that took vitamin A capsules in the preceding six months of the survey were found in age groups 6–11 months and 67 (24.8%) at the age of 12–23 months and the majority 169 (61.9%) were found in age groups 24–59 months. In this study, more than one-third (34.3%) of children received vitamin A supplementation door to door followed by 31.5% by a routine visit to a health facility, 20.88% during the campaign, and 13.2% of sick children supplemented during visiting a health facility for other illness (Fig. 2). Among the total study participants, 198 (42%) children did not receive VAS in the preceding six months of the survey. The main reason described by mothers/caregivers why their child did not take VAS was (45.5%) mothers had no awareness about vitamin A Supplementation, 13.6% fear of toxicity, 28.8% forgot the schedule, 7.6% needs other incentives and 4.5% was due to paternal disapproval (Fig. 3).

### Factors associated with vitamin A supplementation among overall participants

In the bivariable analysis child age, residence, monthly family income, number of under-five children, ANC visit, birth interval, number of ANC visits, birthplace, PNC, nutritional counseling, advice from peers/family about VAS, husbands disapproval about VAS, maternal knowledge about VAS, information about VAS, and history of diarrhea within the last two weeks were included to multivariable analysis at a p-value of ≤ 0.25. After adjustment of these independent variables: monthly family income, postnatal care, frequency of ANC visits, husbands' disapproval of VAS, and information about VAS have been significantly associated with vitamin A supplementation at a *p*-value ≤ 0.05.

Children whose families earn a monthly income greater than 2000 ETB were 2.5 times more likely to receive vitamin A supplementation than those mothers who earn a monthly income less than 2000 ETB [AOR = 2.565, 95% CI(1.631,4.032)]. Our study also showed that mothers who had postnatal care were nearly two folds more likely to receive vitamin A supplementation than those mothers who had not had postnatal care visits [AOR = 1.801, 95% CI (1.158, 2.801)]. Moreover, mothers who had information about vitamin A supplementation were three times more likely to receive VAS than those mothers who did not have information about VAS [AOR = 2.932, 95% CI (1.893, 4,542)]. The current study also found that mothers who were disapproved of by their husbands were 67.6% less likely to receive vitamin A supplementation than those mothers who did not disapprove of their husbands [(AOR = 0.324, 95% CI (0.129, 0.813)]. Finally, mothers who had ≥ 3 ANC follow-ups were two folds more likely to receive vitamin A supplementation than those mothers who had ≤ 2 ANC visits [AOR = 1.882, 95% CI (1.084, 3.266)] (Table 4).

**Table 4:** Multivariable analysis of vitamin A supplementation among children aged 6–59 months in West Azernet Berbere Woreda, South West, Ethiopia, 2021

Variables	Vitamin A supplementation		COR(95%CI)	AOR(95%CI)
	Yes	No		
**Child age (in month)**				
6–11	37	19	1	**1**
12–59	236	179	0.677 (0.377–1.217)	0.829(0.417–1,649)
**Residence**				
Rural	178	161	1	1
Urban	95	37	2.322(1.502–3.590)*	1.474(0.857–2.537)
**Monthly family income**				
≥ 2000	148	122	3.664(2.457–5.493)*	2.565(1.631,4.032)**
< 2000	50	151	1	**1**
**Number of < 5 children**				
≤ 2	258	180	1.720(0.845–3.503)	1.046(0.418–2.615)
3–4	15	18	1	1
**ANC follow-up**				
Yes	261	175	2.859(1.386–5.895)*	1.582(0.874–2.862)
No	12	23	1	1
**Birth interval**				
< 23 month	103	98	1	1
≥ 24 month	170	100	1.617(1.116–2.344)*	1.305(0.818–2.082)
**Number of ANC**				
≤ 2	32	57	1	1
≥ 3	229	118	3.457(2.125–5.623)*	1.882 (1.084, 3.266)**
**Post natal care**				
Yes	156	63	2.857(1.948–4.192)*	1.801 (1.158, 2.801)**
No	117	135	1	1
**Nutrition counseling by Health work**				
Yes	210	121	2.121(1.420–3-168)*	0.842(0.489–1.449)
No	63	77	1	1
**Advice from peers/family about VAS**				
Yes	160	93	1.599(1.106–2.311)*	0.696(0.430–1.128)
No	113	105	1	1
**Husband disapproval**				
Yes	10	24	0.276(0.129–0.591)*	0.324(0.129–0.813)**
No	263	174	1	1
**Maternal knowledge on Vitamin A**				
Poor	149	73	1	1
Good	124	125	0.486(0.334–0.707)*	0.883(0.531–1.470)
**Birth place**				
Health institution	262	166	4.591(2.253–9.359)*	2.316(0.730–7.351)
Home	11	32	1	1
**Information about VAS**				
Yes	185	63	4.505(3.043–6.668)*	2.932 (1.893, 4,542)**
No	88	135	1	1
**Diarrhea in the last two weeks**				
**Yes**	83	88	0.546(0.373–0.799)*	0.674(0.425–1.068)
**No**	190	110	1	1

^*^P value ≤ 0.05 at bivariable logistic regression

^∗ ∗^ *p* value ≤ 0.05 at multivariable logistic regression

## Discussion

This study assessed vitamin A supplementation coverage and associated factors among caregivers having children aged 6–59 months, due to its several benefits for the child. World Health Organization (WHO) recommended vitamin A supplementation two times a year [44].

National coverage estimates are crucial for determining overall program performance, they can also obscure significant variation at the subnational level (i.e., within certain provinces, districts, or communities). These variations are related to both the need for and access to services. To overcome and minimize inequities within countries, it is essential to assess the availability of subnational data and when available, use such disaggregated data to identify areas in need of program strengthening and support. In this regard, this study had its implication.

Despite Ethiopian health sector transformation plan I, II, and the second growth and transformation plan (GTPII), has set ambitious goals to improve equity, coverage, and utilization of essential health services at all levels of the country, our study showed that even though there were some sort of improvement in VAS coverage, inequitable distribution of health outcomes and health services were persistent. Health disparities are still unacceptably wide across different segments of the population and regions. Health indicators vary significantly by socio-economic status, family/husband support, health service-seeking behavior, and information provision.

In this study vitamin, A supplementation coverage was found to be 58% which is higher than the studies carried out in Sidama zone Aleta Chuko woreda (36.2%) [30], EDHS 2016(45%) [45], EMDHS 2019(47%) [23]. Kwazulu-Natal Province, South Africa (34.9%) [46]. Nigeria (41.1%) [47], and India (25%) [48]. The possible reason for this variation may be due to socio-demographic variation, recent advancements in health care delivery, and accessibility of health care services. On the other hand, it is comparable with studies conducted in Wonago district in Southern Ethiopia 59.3% [45], among twenty-three Sub-Saharan African countries 59.4% [38], and Gawadabawa district Sokoto State, Nigeria 61% [49]. However, it is lower when we compared with the studies done in Humbo district (75%) [33], Wolayita(83.1%) [19], Ghana South Dayi district(64.3%) [50], Abuja Nigeria (67%) [51], Guinea (68%) [52]**,** Mali (83%) [53], Bangladesh (68%) [12], (63.5%) [28], and Hegarmanah Village, Jatinangar (92.27%) [54]. The possible justification for variation may be due to differences in socio-demographic and economic characteristics, study setting, and maternal healthcare characteristics.

Based on our findings, family monthly income is found to be an important factor for VAS uptake. Children from families who had to earn high monthly incomes were two folds more likely to receive vitamin A supplementation than those children from families who had to earn less monthly income. Families with favorable socio-demographic characteristics demonstrate good attendance to most public health interventions. This is supported by the study done in Humbo district [33], Aleta Chiko, Sidama [30], South Gondar [29], Nigeria [48, 55], Bangladesh[12, 28], India [56], and selected LMIC in Africa and Asia [57]. The possible justification for this factor might be the richest families can easily access care at a health facility or immunization sites by using the available transportation option. Moreover, they can easily get and consume vitamin A-rich foods, improving their living conditions, and nutritional status. In addition to this, those mothers who earn the highest monthly income may use maternal and child health services regularly, improve uptake of the supplement through advancing access to health information and mitigate economic barters to seeking health care in comparison with their counterparts.

The second pertinent variable associated with VAS was PNC follow-up. Mothers who had a history of postnatal care were two folds more likely to receive VAS than their counterparts**.** This finding is supported by studies carried out in Northwest Ethiopia [58], a systematic review of randomized controlled trials in India [59], and other related literature [60]. A possible explanation could be providing effective and efficient postnatal care services by health care providers will motivate the mother to use postnatal care services frequently. As a result, postpartum supplementation is designed to improve women's vitamin A status and increase the vitamin A content of breast milk. This is meant to protect the mother's vitamin A reserves while addressing one of the fundamental reasons that children become vitamin A deficient—low dietary vitamin A intake from breast milk. Therefore, post-natal VAS improves maternal and infant serum retinol concentrations; maternal and infant liver stores of vitamin A improve breast milk vitamin A concentrations and reduce maternal and infant morbidity.

The current study also found that the number of ANC follow-ups was found to be a determinant of the uptake of vitamin A in their children. Mothers who had ≥3 ANC follow-ups were about two folds more likely to receive vitamin A supplementation than those mothers who had ≤2 ANC visits. This study was congruent with studies conducted in South Gondar[29], Dera District, Northwest Ethiopia[58], analysis of the 2016 EDHS report[16, 27], and Nigeria[48]. This might be due to frequent antenatal care follow-ups allowing pregnant mothers to obtain important health information in wider aspects such as nutritional care and counseling, institutional delivery, exclusive breastfeeding, and immunization. Proper nutritional counseling and support help to enhance nutritional knowledge and appropriate dietary habit of pregnant mothers. These will increase the likelihood of receiving maternal and child health services, particularly vitamin A supplementation. Moreover, attending antenatal care visits creates an opportunity for healthcare workers to provide relevant health information. This may be due to the health information given to pregnant women during antenatal care visits is vital to promote post-delivery health services like vitamin A supplementation. Therefore, contact with health facilities during pregnancy is expected to increase the subsequent use of maternal and child health services.

In this study mothers who were disapproved by their husbands about vitamin, supplementation was 67.6% less likely to receive VAS than those mothers who did not disapprove by their husbands. In this study, only one in five mothers make decisions separately. The majority of the decision was made by either the husband or jointly. This is unsurprising as the society is mainly a patriarchal society where males are heads of households and make decisions for the households This study was supported by the study conducted in Sokoto State, Nigeria [49]. The most common (69%) barrier to the uptake of VAS was found to be fathers' disapproval. The possible reason for this may be connected to the widely held socio-cultural belief that vaccines are harmful to children and are viewed with suspicions, especially its link with Western donor agencies. Fathers' engagement in immunization will boost the family's confidence to fulfill a child's right to be healthy and protect the index child and the community from vaccine-preventable diseases.

Finally, this study identified that mothers who had information about VAS were two times more likely to receive VAS than those mothers who had no information about VAS. This study was supported by studies carried out in the Humbo district in southern Ethiopia [33], and thirteen sub-Saharan African countries [14]. The finding implies that supporting the VAS program with strong information, education, and communication strategies is likely to increase demand and utilization of the supplement. Moreover, providing adequate and timely information for the community members by using the available communication channels helps in improving maternal and child health and sensitizes populations about the importance of vitamin A supplements for young children and broader nutrition messaging. In this study, only 52.65% of mothers have ever heard about vitamin A supplementation. Health workers (44.76%) followed by books and magazines (29.03%) and media (24.6%) were cited as the major source of information for mothers which is similar to studies carried out in Guinea where health agents were cited as the principal sources of VA information followed by radio or television (12%), neighbors (11%), and public criers (7%) [52].In the Humbo district, the leading sources of information were HEWs (97.5%) followed by health development army (HDAs) members (87.8%), health professionals (51.9%), and mass media (20.4%) [33]. The same pattern was also observed in studies done among thirteen sub-Saharan African countries in which community health workers, health facility staffs, and radio messages were key sources of information about VAS [14].

### Limitations of the study

This study cannot ascertain cause and effect relationship since it is a cross-sectional type, the mothers may have memory lapse during data collection due to interaction with other vaccination like polio, the study failed to assess health facility-related factors, and the absence of qualitative study to strengthen the quantitative data by involving the attitude of caregivers related to vitamin A supplementation could be mentioned as a potential limitation of the current study.

## Conclusion and recommendation

Vitamin A supplementation coverage was found to be low compared with the WHO recommendation of 80% and the Health Sector Transformation Plan I (HSTP I) target of 95% and it is strongly associated with family monthly income, postnatal care, husband's disapproval of vitamin A supplementation, antenatal care follow-up, and information about vitamin A supplementation. Based on our findings, it is recommended to improve the monthly income of the household by actively engaging in various income-generating activities, enhance health information dissemination among mothers, particularly those who are underprivileged by using different strategies like local health campaigns, and mass media, advocacy of antenatal, and postnatal follow-up and promote the involvement of males/husband in childhood immunization service.

## Data Availability

The datasets used and/or analyzed during the current study are available from the corresponding author upon reasonable request.

## Abbreviations

AOR: Adjusted Odd Ratio
WHO: World Health Organization
BMI: Body Mass Index
CI: Confidence Interval
SPSS: Statistical Package For Social Sciences
VAS: Vitamin A Supplementation
VA: Vitamin A
HEP: Health Extension Program
EOS: Enhanced Outreach Strategy
CHD: Community Health Day
VAD: Vitamin A Deficiency
COR: Crud Odds Ratio
UNICEF: United Nations International Children’s Fund
EDHS: Ethiopian Demographic and Health Survey
SDG: Sustainable Development Goal
